# Correlation of histologic, imaging, and artificial intelligence features in NAFLD patients, derived from Gd-EOB-DTPA-enhanced MRI: a proof-of-concept study

**DOI:** 10.1007/s00330-023-09735-5

**Published:** 2023-06-26

**Authors:** Nina Bastati, Matthias Perkonigg, Daniel Sobotka, Sarah Poetter-Lang, Romana Fragner, Andrea Beer, Alina Messner, Martin Watzenboeck, Svitlana Pochepnia, Jakob Kittinger, Alexander Herold, Antonia Kristic, Jacqueline C. Hodge, Stefan Traussnig, Michael Trauner, Ahmed Ba-Ssalamah, Georg Langs

**Affiliations:** 1 Department of Biomedical Imaging and Image-Guided Therapy, Medical University of Vienna, Vienna, Austria; 2 Computational Imaging Research Lab, Department of Biomedical Imaging and Image-Guided Therapy, Medical University of Vienna, Vienna, Austria; 3 Department of Pathology, Medical University of Vienna, Vienna, Austria; 4 Division of Gastroenterology and Hepatology, Department of Medicine III, Medical University of Vienna, Vienna, Austria; 5 Department of Biomedical Imaging and Image-Guided Therapy, General Hospital of Vienna (AKH), Medical University of Vienna, Waehringer Guertel 18-20, 1090 Vienna, Austria

**Keywords:** Non-alcoholic fatty liver disease, Magnetic resonance imaging, Gadolinium ethoxybenzyl DTPA, Artificial intelligence, Deep learning

## Abstract

**Objective:**

To compare unsupervised deep clustering (UDC) to fat fraction (FF) and relative liver enhancement (RLE) on Gd-EOB-DTPA-enhanced MRI to distinguish simple steatosis from non-alcoholic steatohepatitis (NASH), using histology as the gold standard.

**Materials and methods:**

A derivation group of 46 non-alcoholic fatty liver disease (NAFLD) patients underwent 3-T MRI. Histology assessed steatosis, inflammation, ballooning, and fibrosis. UDC was trained to group different texture patterns from MR data into 10 distinct clusters per sequence on unenhanced T1- and Gd-EOB-DTPA-enhanced T1-weighted hepatobiliary phase (T1-Gd-EOB-DTPA-HBP), then on T1 in- and opposed-phase images. RLE and FF were quantified on identical sequences. Differences of these parameters between NASH and simple steatosis were evaluated with *χ*^2^- and *t*-tests, respectively. Linear regression and Random Forest classifier were performed to identify associations between histological NAFLD features, RLE, FF, and UDC patterns, and then determine predictors able to distinguish simple steatosis from NASH. ROC curves assessed diagnostic performance of UDC, RLE, and FF. Finally, we tested these parameters on 30 validation cohorts.

**Results:**

For the derivation group, UDC-derived features from unenhanced and T1-Gd-EOB-DTPA-HBP, plus from T1 in- and opposed-phase, distinguished NASH from simple steatosis (*p* ≤ 0.001 and *p* = 0.02, respectively) with 85% and 80% accuracy, respectively, while RLE and FF distinguished NASH from simple steatosis (*p* ≤ 0.001 and *p* = 0.004, respectively), with 83% and 78% accuracy, respectively. On multivariate regression analysis, RLE and FF correlated only with fibrosis (*p* = 0.040) and steatosis (*p* ≤ 0.001), respectively. Conversely, UDC features, using Random Forest classifier predictors, correlated with all histologic NAFLD components. The validation group confirmed these results for both approaches.

**Conclusion:**

UDC, RLE, and FF could independently separate NASH from simple steatosis. UDC may predict all histologic NAFLD components.

**Clinical relevance statement:**

Using gadoxetic acid–enhanced MR, fat fraction (FF > 5%) can diagnose NAFLD, and relative liver enhancement can distinguish NASH from simple steatosis. Adding AI may let us non-invasively estimate the histologic components, i.e., fat, ballooning, inflammation, and fibrosis, the latter the main prognosticator.

**Key Points:**

*• Unsupervised deep clustering (UDC) and MR-based parameters (FF and RLE) could independently distinguish simple steatosis from NASH in the derivation group.*

*• On multivariate analysis, RLE could predict only fibrosis, and FF could predict only steatosis; however, UDC could predict all histologic NAFLD components in the derivation group.*

*• The validation cohort confirmed the findings for the derivation group.*

**Supplementary information:**

The online version contains supplementary material available at 10.1007/s00330-023-09735-5.

## Introduction

Non-alcoholic fatty liver disease (NAFLD) has become a significant public health problem as its incidence continues to increase [1, 2]. NAFLD comprises simple steatosis, with relatively low liver-related morbidity, and non-alcoholic steatohepatitis (NASH), which may lead to progressive hepatic dysfunction and liver-related mortality [3]. While simple steatosis typically improves with lifestyle changes, NASH may require additional pharmacotherapy [1, 2]. The sequelae of NASH, i.e., end-stage liver cirrhosis, liver failure, hepatocellular carcinoma (HCC), and/or eventual liver transplantation, can be mitigated through early diagnosis and management [4, 5].

Currently, NASH is still routinely diagnosed by liver biopsy, an invasive procedure which increases the risk of bleeding in patients already prone to coagulopathy. Thus, patient acceptance is poor, restricting its utility for long-term monitoring. Further limitations include sampling errors due to uneven distribution of steatosis and high inter-observer variability [6, 7]. Moreover, universal liver biopsy is not feasible in a high-prevalence disease such as NAFLD. In addition, serum markers are largely nonspecific and conventional imaging, including US, CT, and gadolinium chelate-enhanced MRI, cannot differentiate between NASH and simple steatosis [8, 9]. Thus, a non-invasive diagnostic test, with both high sensitivity and specificity for detection and monitoring of NASH, is urgently needed [10].

Multiparametric magnetic resonance imaging (MRI) with its ability to quantify proton density fat fraction (PDFF), using a gamut of techniques such as dual-echo chemical shift imaging (CSI), i.e., in- and opposed-phase [11] [12], multi-echo technique, or MR proton spectroscopy (MRS) [13], as well as detecting fibrosis and inflammation with MR elastography [14], has emerged as a powerful tool.

Gd-EOB-DTPA-MRI, initially used to detect and characterize focal liver lesions, such as HCC complicating NAFLD, has been shown to distinguish between simple steatosis and NASH from the calculated relative liver enhancement (RLE) [15]. Also, CSI was able to differentiate between both entities using the fat fraction (FF) [16]. Furthermore, artificial intelligence (AI), including deep learning, may shed light on the imaging features of NAFLD. Recently, an unsupervised predictive texture discovery, proposed by Perkonigg et al, was introduced [17]. This approach is based on the deep clustering networks (DCN) [18] and uses random forests to link the histologically-relevant information to the texture patterns extracted by this approach [19]. Therefore, the aim of this study was to investigate in a derivation group whether a hybrid unsupervised and supervised deep learning approach could identify predictive patterns that could differentiate simple steatosis from NASH using the CSI technique, as well as unenhanced T1 and Gd-EOB-DTPA-MR images in the hepatobiliary phase (T1-Gd-EOB-DTPA-HBP). Furthermore, we compared the ability of UDC with that of RLE and FF, all data derived from identical MR sequences, to distinguish between NASH and simple steatosis in NAFLD patients. Histopathology was used as the gold standard. After identifying simple steatosis vs NASH predictors in the derivation group, we applied this model to a validation group.

## Materials and methods

### Patients

Written informed consent was obtained from all patients and the study protocol approved by the local ethics committee for this single-center study. Whereas the derivation cohorts were enrolled prospectively, the validation cohorts, imaged on another scanner with different software and exam parameters, were gathered retrospectively.

Patients with clinical features suspicious for fatty liver on ultrasound and elevated serum levels of aspartate and alanine aminotransferase were recruited from the Division of Gastroenterology and Hepatology of our tertiary academic institution. Inclusion criteria included histologic proof of simple steatosis or NASH and use of a standardized complete Gd-EOB-DTPA-enhanced MR protocol. Exclusion criteria were age < 18 years, pregnancy, alcohol consumption of ≥ 20 g/day, presence of hepatitis B and C infection, autoimmune liver diseases, hemochromatosis, Wilson’s disease, α-1 antitrypsin deficiency, toxic liver diseases, primary biliary cirrhosis, and primary sclerosing cholangitis, respectively, according to American and European current guidelines [1, 2]. There were 49 derivation and 30 validation patients. We excluded three derivation-group patients, two with incomplete MRI and one who refused biopsy. The final derivation cohort included 46 patients and 30 validation patients, all with complete MRI and histology reports.

### Reference standard: biopsy and histopathological analysis

All liver biopsy specimens were evaluated by an experienced pathologist using the Steatosis Activity Fibrosis (SAF) scoring system as the gold standard [20], including steatosis grade (mild, moderate, and severe), and two of these three features: (1) necro-inflammation with mononuclear cells and/or polymorphonuclear leukocytes, (2) ballooning degeneration of hepatocytes, and (3) perisinusoidal and/or bridging fibrosis.

### Blood markers

For blood markers, we considered common biochemical parameters, including levels of total bilirubin, aspartate aminotransferase, alanine aminotransferase, alkaline phosphatase, g-glutamyl transpeptidase, triglycerides, high-density lipoprotein cholesterol, and glucose. In all patients, the serum markers were measured in the same laboratory within 1 week of MR imaging. Furthermore, we used the FIB-4 score, the NAFLD Fibrosis Score (NFS), the ALBI score, and the APRI score as established non-invasive biomarkers for accurate stratification of patients at higher risk of NASH and advanced fibrosis.

### MRI protocol

All derivation-group MR examinations were performed on a 3-T scanner (Magnetom Trio, A Tim) and all validation-group exams were done on a 3-T (Magnetom Prisma Fit) Siemens Healthineers. The MRI protocol included a chemical shift imaging (CSI) technique, with in-phase and opposed-phase transverse T1-weighted, dual gradient-echo sequence pre-contrast media. Furthermore, unenhanced and dynamic contrast-enhanced, three-dimensional, breath-hold, T1-weighted spoiled gradient-echo volumetric (VIBE) sequences, including the hepatobiliary phase, i.e., 20 min after CM injection, diffusion-weighted images (DWI), and conventional T2-weighted images, were acquired. A standard dose of Gd-EOB-DTPA (0.025 mmol/kg; Primovist® in Europe and Eovist® in the USA; Bayer Healthcare, Berlin, Germany) was administered as a bolus intravenously, for all patients of both groups using a power injector at a rate of 1.0 mL/s, immediately followed by a 20-mL saline flush. MR acquisition parameters are given in Table 1 and Table 1S.

**Table 1:** Derivation group. MR protocol with exam parameter

Sequences	Slice orientation	Matrix	Voxel (mm)	FOV (mm)	SL (mm)	Phase direction	TR (ms)	TE (ms)	FA (degree)	Time (s)
GRE T1 (2D flash) in-phase*	Axial	320 × 320	0.3 × 0.3 × 1.7	410	5	AP	130 – 140	2.46	70	17
GRE T1 (2D flash) opposed-phase*	Axial	320 × 320	0.3 × 0.3 × 1.7	410	5	AP	130 – 180	3.69	70	17
T1 VIBE FatSat unenhanced*	Axial	512 × 320	1.1 × 1.1 × 1.7	430	1.7 – 2	AP	2.67	0.97	13	16
T1 VIBE FatSat gadoxetic-enhanced (arterial and portal-venous)	Axial	512 × 320	1.1 × 1.1 × 1.7	430	1.7 – 2	AP	2.67	0.97	13	16 × 3
T1 VIBE FatSat 5 min post contrast (transitional)	Axial	512 × 320	1.1 × 1.1 × 1.7	430	1.7 – 2	AP	2.67	0.97	13	16
DWI TSE-EP/ADC	Axial	384 × 288	1.5 × 1.5 × 5	400	5	AP	5000–	73	90	133
T2 HASTE fs T2 HASTE	Axial Coronal	320 × 256 256 × 256	1.1 × 1.1 × 5 1.6 × 1.6 × 5	400 400	5 5	AP RL	1800 805	150 78	150 150	47 120
T1 VIBE FatSat 20 min post contrast (HBP)*	Axial	512 × 320	1.1 × 1.1 × 1.7	430	1.7 – 2	AP	2.67	0.97	13	16
T1 VIBE FatSat 20 min post contrast (HBP)	Coronal	232 × 256	1.3 × 1.3 × 2.2	500	2.0	RL	2.6	0.92	13	16

*HASTE*, half-Fourier acquisition single-shot turbo spin echo imaging; *DWI TSE-EP/ADC*, diffusion-weighted imaging turbo spin echo-echo-planar; *MRCP*, magnetic resonance cholangiopancreatography; *MIP*, maximum intensity projection; *GRE*, gradient echo; *VIBE*, volumetric interpolated breath-hold examination, *FOV*, field of view; *Voxel*, voxel size; *SL*, slice thickness; *TR*, repetition time; *TE*, echo time; *FA*, flip angle; *Time*, acquisition time

^*^Evaluated for the study

### Image analysis

**Fig. 1:**
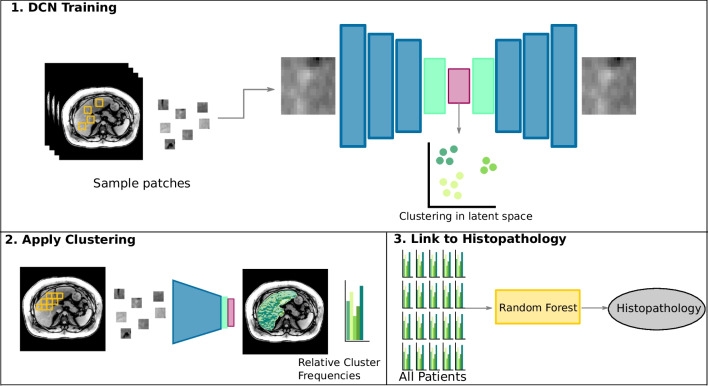
A schematic showing the UDC (unsupervised deep clustering) analysis of liver imaging texture features on axial unenhanced and Gd-EOB-DTPA-enhanced T1-weighted hepatobiliary phase (T1-GA-HBP) images, as well as dual echo in- and opposed-phase images (CSI) to differentiate between NASH and simple steatosis. (1) In the course of DCN training, the model studies all images in the training set and establishes various prototype clusters based on the spectrum of textures represented on the images. (2) During application on a cohort, the model translates imaging data into prototype clusters. Quantification of their relative abundance on each image results in (3) a histogram that serves as a feature representation of that liver MR section. A prediction model infers histopathology parameters from the features [17]

*Computational image analysis and UDC* had two main steps combining supervised and unsupervised machine learning, as follows [17]:

• First, in a pre-processing step, the liver was automatically segmented on MR sequences in all image volumes using a convolutional neural network architecture called U-Net which is particularly well-suited for image segmentation tasks [21].

• Then, unsupervised machine learning, using a combined deep learning and clustering method, identified a set of image patterns frequent on liver MRI across NAFLD patients. For our 46 NAFLD patients, 50,000 2D patches in the axial orientation were randomly extracted [22]. The clusters of every liver were also linked to the histological target variables for that liver.

• Then an autoencoder network that had been trained to reconstruct low-dimensional input accurately used three convolutional layers and three upsampling operations to rebuild the liver images in the latent space.

• Simultaneously, the DCN method assigned patches with similar appearances in this latent space into 10 distinct clusters.

• Lastly, we had the trained network use a sliding window to parse (i.e., search) the entire axial liver slice of all 46 NAFLD patients. At each position, it extracted, processed, and assigned the patch to one of the 10 clusters derived during the training. The UDC signature of each liver was the relative proportion of that liver image that belonged to each of the 10 clusters, i.e., a histogram. An overview of the method is illustrated in Fig. 1.

• Then, we created 46 × 3 UDC, one for each MRI sequence of each cohort: unenhanced T1-, T1-Gd-EOB-DTPA-HBP, and [unenhanced T1-in-phase and unenhanced T1-opposed phase]. To combine information from unenhanced T1- and Gd-EOB-DTPA-HBP scans, we created a 10-component UDC signature for each, and combined them by concatenation resulting in a 20-component UDC signature for each patient. UDC signatures for T1 in- and opposed-phase images were calculated independently from the Gd-EOB-DTPA-enhanced images and resulted in an additional 10-dimensional feature vector per patient.

• In the second step, the UDC signatures of liver scans were used as feature vectors to perform supervised machine learning with a Random Forest regression model [19]. Then, those feature vectors were tested to see if and how accurately they could predict histologically-relevant features and grades of steatosis, inflammation, fibrosis, and ballooning to classify the patient as simple steatosis or NASH. In other words, this cross-validation tested the model’s performance.

*Conventional MRI quantification* analysis used signal intensity (SI) measurements performed on a commercially available workstation (PACS system, AGFA-Healthcare, version 5.2) by two independent observers: a fellowship-trained radiologist with more than 8 years of experience (N.B.) in abdominal MR imaging, and a technologist with 3 years’ MR experience (R.F.). Both observers were blinded to patients’ clinical history, laboratory data, and histopathology characteristics.

**Fig. 2:**
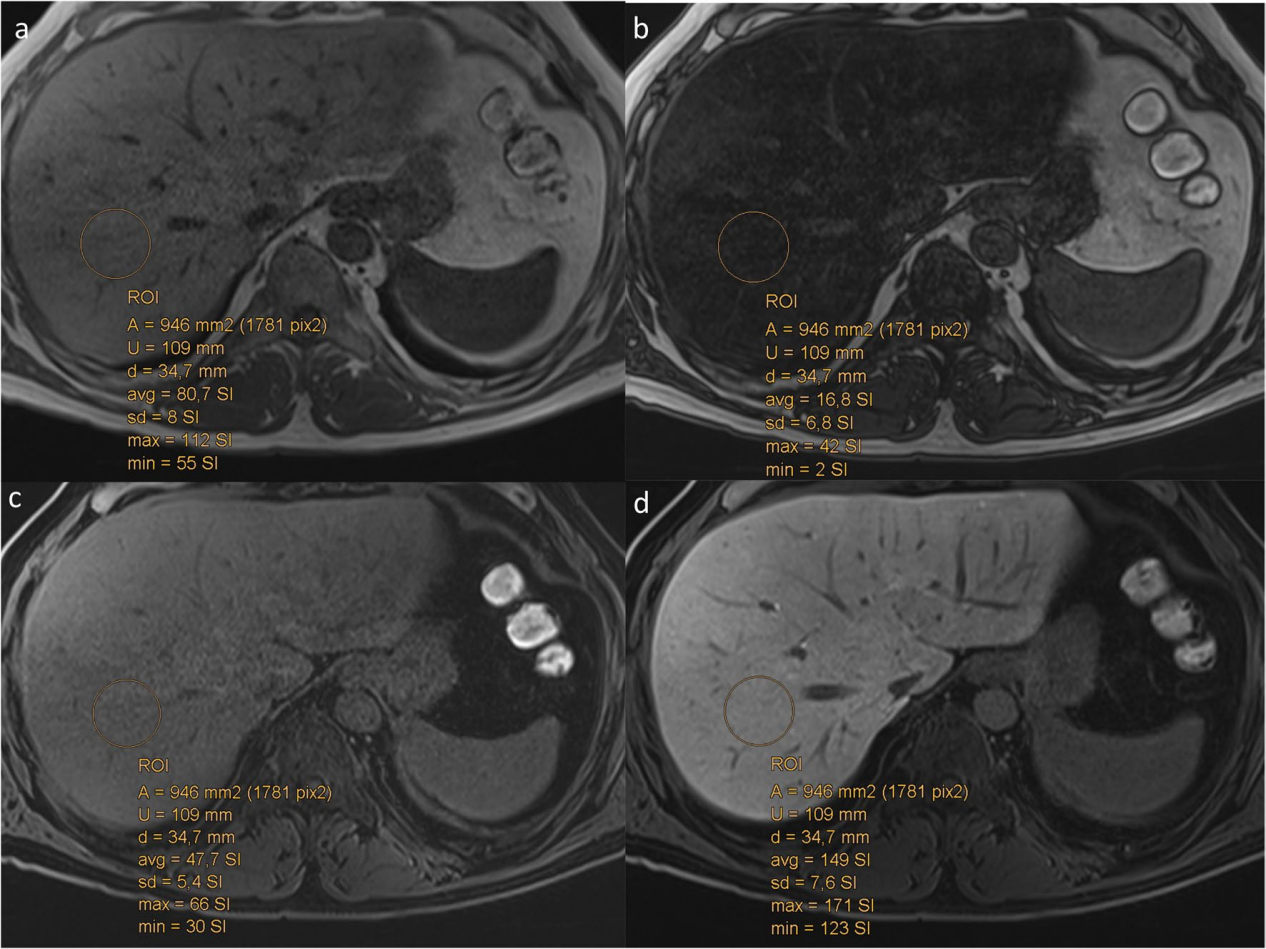
**a, b** Axial chemical shift images (CSI). **a, b** Dual-echo, in- and opposed-phases, showing a diffuse loss of liver signal intensity. The fat fraction (FF) calculated as the mean of all nine segments (i.e., seg 4 a and b) of the liver according to the formula: [(SIin-SIopposed)/2 × SIin] × 100. SIin and SIopposed were the liver parenchyma signal intensity on in-phase or opposed-phase images, respectively. **c** Axial unenhanced (PreSI), and **d** contrast-enhanced (T1-GA-HBP) images obtained 20 min after Gd-EOB-DTPA injection in the hepatobiliary phase (HBP) (PostSI) showing the calculation of the RLE according to the formula: Relative Liver Enhancement (RLE) = (PostSI-PreSI)/PreSI

•The liver parenchymal SI was measured on unenhanced (PreSI), then on contrast-enhanced images obtained 20 min after contrast medium administration (PostSI) [15]. Measurements were performed by positioning nine separate circular regions of interest (ROIs) ≥ 1 cm in diameter in each Couinaud liver segment, including segments 4a and b separately (Fig. 2). ROIs were drawn to avoid vascular motion and abdominal wall artifacts and were positioned far from visible vascular and biliary structures. Liver SIs were calculated as the relative enhancement reported on the unenhanced images, according to the formula: Relative Liver Enhancement (RLE) = (PostSI-PreSI)/PreSI, as previously described in detail [15].

•The hepatic fat fraction (FF) was calculated by both radiologists independently. Again, they placed the ROIs as described above in all liver segments on the in- and opposed-phase sequences. Liver fat was quantified as follows: [(SIin-SIopp)/2 × SIin] × 100 as the percentage of relative signal intensity loss of the liver parenchyma on opposed-phase images. SIin and SIopp were liver parenchyma signal intensity on in-phase or opposed-phase images, respectively [23].

•Finally, we calculated the average liver SI for RLE and FF by adding the mean signal intensity of all Couinaud segments for RLE and FF, respectively.

### Statistical analysis

Categorical variables are presented as numbers and percentages, and continuous variables as means and standard deviations. Differences between NASH and simple steatosis were evaluated by the *χ*^2^ test for categorical data, and differences in continuous data between both groups were assessed using Student’s *t*-test. Mean RLE or mean FF was first tested with univariate and then with multiple regression analysis to see whether there was an association with NAFLD’s histologic features and to identify independent imaging predictors to distinguish NASH from simple steatosis. For UDC signatures, we used a Random Forest classifier to link those features to histology and evaluate their predictive values. To assess the diagnostic performance of the two methods (UDC features and conventional MRI quantification methods, i.e., mean FF and mean RLE) to accurately separate NASH from simple steatosis, a receiver-operating characteristic (ROC) curve analysis was performed and optimal cutoff values were chosen by using a common optimization step that maximized the Youden index for predicting which patients had NASH. Subsequently, sensitivity, specificity, accuracy, positive predictive values (PPV), and negative predictive values (NPV) for the appropriate cutoffs and area under the curve (AUC) for both methods were calculated. The inter-rater variability was assessed by two-way mixed intraclass correlation coefficient (ICC) with absolute agreement [24]. The DeLong test was performed to compare the AUC for the combined UDC, RLE, and FF features for the derivation and validation groups [25]. All statistical analyses were performed for the derivation and validation in SPSS 25.0 (SPSS Inc) or Python v3.7.0. Statistical significance was set at a *p* value of less than 0.05.

## Results

### Derivation group

#### Characteristics

Forty-six patients prospectively enrolled, consisting of *M* = 29 (63%), mean age of 49 years (range, 18–78 years). The mean age for women was 44.62 years (range, 18–64 years), and for men 51.52 years (range, 23–81 years). Histologically, 28 (61%) met the criteria for NASH, leaving 18 classified as simple steatosis.

There were more men than women in the NASH group, but the differences between gender, age, and BMI were not statistically significant (Table 2). The interval between MRI and liver biopsy was 1 to 3 days.

**Table 2:** Derivation group. Anthropometric, clinical, and laboratory characteristics of 46 patients of the two groups of NAFLD (simple steatosis, and NASH)

Parameter	Simple steatosis (18 patients)	NASH (28 patients)	*p* value
Age (years)			
All patients (*n* = 46)	47.6 ± 15.4 (18.1–78.6)	50.1 ± 11.4 (25.2–69.3)	0.529
Men (*n* = 29)	50.7 ± 15.40	48.17 ± 12.34	0.854
Women (*n* = 17)	43.45 ± 4.11	53.79 ± 8.19	0.553
Body mass index (kg/m^2^)	29.2 ± 4.7 (19.7–37.8)	29.8 ± 4.5 (22.5–41.1)	0,258
Alanine aminotransferase (ALT) (U/L)	52.1 ± 23.9 (19–218)	87.6 ± 57.1 (22–693)	0.772
Aspartate aminotransferase (AST) (U/L)	31.7 ± 7.9 (17–294)	62.3 ± 60.6 (19–474)	0.816
g-glutamyl transpeptidase (GGT) (U/L)	67.4 ± 44.4 (25–459)	206.9 ± 329.3 (19–1551)	0.154
Alkaline phosphatase (ALT) (U/L)	74.6 ± 27.8 (22–218)	91.9 ± 42.5 (19–693)	0.672
Total proteins (g/ L)	70.2 ± 3.5 (60–81)	71.7 ± 4.2 (62–80)	0.162
Albumin (g/L)	43.4 ± 3.1(25–51)	45.7 ± 4.3 (36–52)	0.084
Total bilirubin (mg/dL)	0.61 ± 0.31 (0–1)	0.76 ± 0.23 (0–2)	0.061
Triglycerides (mg/dL)	134.4 ± 101.7 (0–368)	168.3 ± 106.2 (55–522)	0.106
HDLC (mg/dL)	51.8 ± 10.9 (32–65)	47.4 ± 12.3 (31–74)	0.481
Platelets (/mm^3^)	235.1 ± 73.5 (136–354)	200.3 ± 76.1 (88–450)	0.186
Glucose (mg/dL)	87.8 ± 44.5 (0–113)	114.7 ± 30.9 (80–202)	0.082
AST/ALT ratio	0.69 ± 0.17 (0.36–0.94)	.081 ± 43 (0.33–1.85)	0.288
APRI_limit42male_35female -Score	0.72 ± 0.12(0.12–0.39)	0.76 ± 0.69 (0.16–0.39)	0.707
APRI_limit50male_35female -Score	0.68 ± 0.10 (0.10–0.44)	0.82 ± 0.80 (0.14–0.33)	0.899
ALBI -Score	− 3.04 ± 2.78 (− 3.70 to 2.68)	− 3.17 ± 0.37 (− 3.77 to 2.38)	0.323
NFS-Score	− 26.36 ± 10.85 (− 42.49 to 11.90)	− 15.09 ± . (− 45.04 to 21.05)	**0.049**
Fib-4-Score	1.05. ± 48 (0.49–0.21)	1.75 ± 1.48 (0.38–0.64)	0.071

Data are means and standard deviations with ranges in parentheses, except where indicated otherwise. To convert from units per liter to micrograms per liter, multiply by 0.0167. To convert from milligrams per deciliter (for bilirubin) to micromoles per liter, multiply by 17.104. To convert from milligrams per deciliter (for triglycerides) to millimoles per liter, multiply by 0.0113. To convert from milligrams per deciliter (for high-density lipoprotein cholesterol) to millimoles per liter, multiply by 0.0259. To convert from milligrams per deciliter (for glucose) to millimoles per liter, multiply by 0.0555

*Abbreviations*: *APRI*, aspartate aminotransferase to platelet ratio index; *ALBI*, albumin-bilirubin score; *NFS*, NAFLD Fibrosis Score; *FIB-4 score*, Fibrosis index based on 4 factors

FIB-4 = Age (years)Å ~ AST (U/L)/[PLT(109/L)Å ~ ALT1/2 (U/L)]

NAFLD fibrosis score =  − 1.675 + 0.037 Å ~ age (year) + 0.094 Å ~ BMI (kg/m^2^) + 1.13 Å ~ IFG/diabetes (yes = 1, no = 0) + 0.99 Å ~ AST/ALT ratio—0.013 Å ~ platelet count (Å ~ 109/L)—0.66 Å ~ albumin (g/dL)

ALBI score = (log10 bilirubin [μmol/L] × 0.66) + (albumin [g/L] ×  − 0.0852)

APRI score = [(AST/upper limit of the normal AST range) × 100]/Platelet

The liver enzymes were generally higher in NASH than in simple steatosis patients. However, the difference was not statistically significant in the majority of these data (Table 2). Established clinical scores, including AST/ALT ratio, and APRI, ALBI, NFS, and Fib-4 scores, were also higher in NASH patients. However, only the NFS score reached statistical significance (Table 2).

**Table 3:** Derivation group. Histological characteristics of NAFLD patients according to SAF score

Histology parameters	Simple steatosis patients (*n* = 18)	NASH patients (*n* = 28)	*p*
Steatosis grade			***p < 0.002***
1 (5–33%)	11 (61.1%)	9 (32.1%)	
2 (34–66%)	4 (22.2%)	5 (17.9%)	
3 (> 66%)	3 (16.7%)	14 (50.0%)	
Lobular inflammation activity			***p*** ** < 0.0001**
0 (none)	13 (72.2%)	0 (0.0%)	
1 (≤ 2 foci per × 20 magnification)	5 (27.8%)	18 (64.3%)	
2 (> 2 foci per × 20 magnification)	0 (0%)	10 (35.7%)	
Ballooning activity			***p*** ** < 0.005**
0 (none)	14 (77.8%)	0 (0.0%)	
1 (slight)	3 (16.7%)	21 (75.0%)	
2 (clear)	1 (5.6%)	7 (25.0%)	
Fibrosis			***p*** ** = 0.001**
0	9 (50.0%)	1 (3.6%)	
1a, b, c	4 (22.2%)	11 (39.3%)	
2	4 (22.2%)	4 (14.3%)	
3	0 (0%)	8 (28.6%)	
4	1 (5.6%)	4 (14.3%)	

Data are numbers of patients and numbers in parentheses are percentages, except where indicated otherwise, *p *< 0.05 indicates significance

*NAFLD*, non-alcoholic fatty liver disease; *SAF*, steatosis activity fibrosis; *NASH*, non-alcoholic steatohepatitis

All 4 values in the last column are significant (marked in bold) since * p *< 0.05

The final liver histology diagnosis and the distribution of fatty infiltration, lobular inflammation, ballooning, and fibrosis stage according to the SAF score (i.e., S ≥ 1, A ≥ 1 + ≥ 1, any F score for NASH) were used as the gold standard (Table 3). The NASH group had a significantly higher number of patients with increased lobular inflammation (*p* < 0.0001), steatosis (*p* = 0.002), and ballooning (*p* = 0.005), as well as fibrosis (*p* = 0.001), compared to those with simple steatosis.

### Results of liver segmentation

The U-Net used for liver segmentation was trained on the derivation liver cohort. We randomly sampled 7 of the 46 patients and created ground truth labels for the evaluation of the segmentation accuracy on both T1-Gd-EOB-DTPA-HBP and unenhanced T1 sequences. We found an increased accuracy for T1-Gd-EOB-DTPA-HBP (Dice: 0.960, recall: 0.945, precision: 0.976) compared to unenhanced T1 sequence (Dice: 0.897, recall: 0.961, precision: 0.842).

### Results of UDC

In the derivation group (*p* ≤ 0.001) overall, we were able to find features that distinguished NASH from simple steatosis using Student’s *t*-test (Table 4). The results derived from unenhanced T1- and T1-Gd-EOB-DTPA MRI in the hepatobiliary phase (T1-Gd-EOB-DTPA-HBP) for fibrosis, steatosis, lobular inflammation, and hepatocyte ballooning using Random Forest regression were calculated. Using the UDC in the derivation group, we could predict variables differentiating between low- (grade 0, 1) and high-grade steatosis (*p* < 0.001), low- (grade < 3) and high-grade fibrosis (*p* = 0.0005), and also gradations of lobular inflammation (*p* = 0.001) and ballooning (*p* = 0.04).

**Table 4:** Derivation group. MR imaging and UDC parameters demonstrating the differences between simple steatosis and NASH of 46 patients with NAFLD according to the SAF score for both readers (R1 and R2) using the *t*-test

Parameter	Simple steatosis 18 patients R1	NASH 28 patients R1	^†^*p* value	Simple steatosis 18 patients R2	NASH 28 patients R2	^†^*p* value	ICC
UDC (unenhanced T1 and Gd-EOB-DTPA-T1-HBP)	0.36 ± 0.23	0.71 ± 0.24	** < 0.001**				
UDC (CSI, in- and opposed-phase)	0.48 ± 0.27	0.67 ± 0.27	**0.02**				
Mean signal intensity unenhanced T1	285.73 ± 52.76	266.54 ± 43.15	0.539	251.16 ± 66.14	244.33 ± 46.19	0.266	0.813
Mean signal intensity Gd-EOB-DTPA-T1-HBP	761.48 ± 240.40	528.12 ± 83.67	**0.001**	652.62 ± 22.15	454.61 ± 19.11	**0.044**	0.958
Mean relative liver enhancement (RLE)	1.58 ± .52	1.01 ± .27	** < 0.001**	1.49 ± .43	1.12 ± .41	**0.005**	0.784
FF (PDFF/CSI, in- and opposed-phase)	24.53 ± 6.83	50.56 ± 19.01	**0.004**	22.82 ± 17.52	39.82 ± 19.26	**0.005**	0.980

^*^Data are means with standard deviations

^†^If the *p* value was less than the conventional level of .05, the corresponding variable was statistically significant and is written in bold type

*RLE*, relative liver enhancement; *FF*, fat fraction; *CSI*, chemical shift imaging dual echo: in-phase and out-of-phase; *UDC* (unenhanced T1 and HBP): unsupervised deep clustering derived from unenhanced T1 and T1, 20 min after injection of Gd-EOB-DTPA acid in the hepatobiliary phase (HBP); *UDC (CSI, in- and opposed-phase)*, unsupervised deep clustering derived from chemical shift imaging (in-phase and out-of-phase)

All 4 values in the last column are significant since *p *< 0.05

**Fig. 3:**
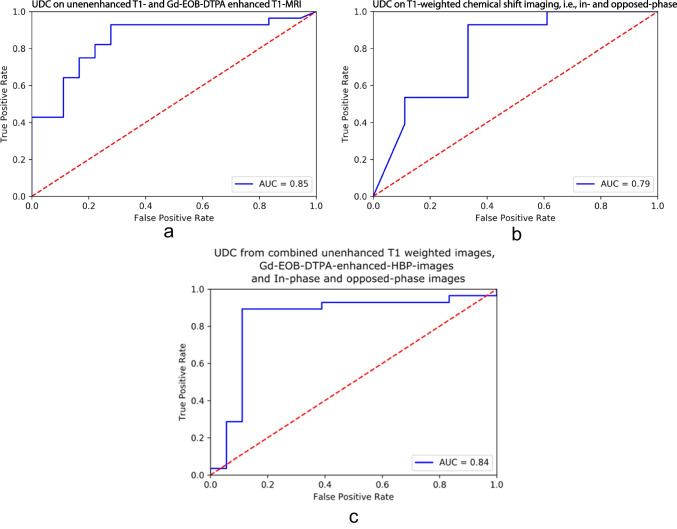
ROC curves showing the random forest-based diagnostic performance of UDC for differentiating NASH from simple steatosis, based on histology, using (**a**) unenhanced and T1-GA-HBP; (**b**) CSI, i.e., in-phase and opposed-phase; and (**c**) combined unenhanced, T1-Gd-EOB-DTPA-HBP and CSI. **a** The random forest classifier, based on (a) unenhanced and T1-GA-HBP, was able to differentiate NASH from simple steatosis patients with an accuracy of 85.2% [AUROC = 0.854], a sensitivity of 89.2%, a specificity of 72.2%, a PPV of 83.3%, and an NPV of 81.3%. **b** The Random Forest classifier, based on (**b**) in- and opposed-phase (CSI), was able to differentiate NASH from simple steatosis patients with an accuracy of 80.4% [AUROC = 0.792], a sensitivity of 89.3%, a specificity of 66.6%, a PPV of 80.6%, and an NPV of 80.0%. **c** The Random Forest classifier, based on unenhanced T1- and T1-Gd-EOB-DTPA-HBP combined with CSI, was able to differentiate NASH from simple steatosis patients with an accuracy of 78.3% [AUROC = 0.84], a sensitivity of 75.0%, a specificity of 83.3%, a PPV of 87.5%, and a NPV of 68.2%

Furthermore, Random Forest classifier was able to differentiate NASH from simple steatosis patients with an accuracy of 85.2% [AUROC = 0.854 (95% CI: 0.76–0.98)], a sensitivity of 89.2%, a specificity of 72.2%, a PPV of 83.3%, and a NPV of 81.3% (Fig. 3a).

In the derivation group, UDC signatures derived from CSI (T1-weighted chemical shift imaging, i.e., in- and opposed-phases) were able to differentiate between NASH and simple steatosis using Student’s *t*-test (*p* = 0.02) (Table 4). Using Random Forest regression, we could distinguish only between low- and high-grade of steatosis (*p* = 0.02) and inflammation (*p* = 0.01). UDC based on CSI failed to capture features that could reliably separate the various grades of fibrosis (*p* = 0.13) or hepatocyte ballooning (*p* = 0.65).

Random Forest classifier allowed us to distinguish NASH from simple steatosis patients with an accuracy of 80.4% [AUROC = 0.792 (95%CI 0.76–0.98)], a sensitivity of 89.3%, a specificity of 66.6%, a PPV of 80.6%, and a NPV of 80.0%. The ROC curve is depicted in Fig. 3b.

The Random Forest classifier, based on unenhanced T1- and T1-Gd-EOB-DTPA-HBP combined with CSI, was able to differentiate NASH from simple steatosis patients with an accuracy of 78.3% [AUROC = 0.84], a sensitivity of 75.0%, a specificity of 83.3%, a PPV of 87.5%, and a NPV of 68.2%. The combined ROC curve is depicted in Fig. 3c.

### Results of MR-derived measurements (RLE and FF)

MRI parameters, derived from the same images as those used in the UDC, i.e., unenhanced T1- and Gd-EOB-DTPA-enhanced MRI (T1-Gd-EOB-DTPA-HBP) and CSI sequences, were significantly different in NASH compared to simple steatosis patients for both readers. Moreover, there was excellent inter-reader agreement for these measurements, with high ICC (0.8–0.9) values (Table 4).

Univariate and multivariate analyses of the relationship between RLE, FF, and histopathologic parameters are summarized in Table 5. In the univariate analysis, RLE was negatively correlated with the degree of liver steatosis (Beta =  − 0.422, *p* = 0.004), lobular inflammation (Beta =  − 0.408, *p* = 0.005), and degree of fibrosis (Beta =  − 0.500, *p* ≤ 0.001), but not with the activity score for ballooning (Beta =  − 0.282, *p* = 0.059).

**Table 5:** Derivation group. Correlation of conventional MR parameters using RLE/FF and histologic parameters according to univariate and multiple regression analyses for reader 1

Parameter	Univariate					Multivariate				
RLE	*B*	*p* value	Beta	95% CI		*B*	*p* value	Beta	95% CI	
Steatosis	− 0.171	0.004	− 0.422	− 0.283	− 0.059	− 0.099	0.052	− 0.235	− 0.202	0.012
Inflammation	− 0.234	0.005	− 0.408	− 0.393	− 0.075	− 0.144	0.073	− 0.251	− 0.292	0.004
Ballooning	− 0.159	0.059	− 0.281	− 0.325	0.006					
Fibrosis	− 0.164	< 0.001	− 0.500	− 0.251	− 0.078	− 0.132	**0.040**	− 0.397	− 0.213	− 0.048
FF	B	*p* value	Beta	95% CI		*B*	*p* value	Beta	95% CI	
Steatosis	13.776	< 0.001	0.733	9.887	17.666	13.600	**< 0.001**	0.723	9.9382	17.818
Inflammation	9.757	0.012	0.367	2.245	17.269					
Ballooning	2.775	0.485	0.105	− 5.173	10.724					
Fibrosis	1.055	0.647	0.069	− 3.564	5.674					

If the *p* value is less than the conventional level of .05, the corresponding variable contributes significantly to the prediction of the dependent variable (RLE or FF). In multiple regression analysis, only liver fibrosis was significantly associated with the relative enhancement measurements (RLE) and only steatosis was significantly associated with fat fraction (*FF*)

*RLE*, relative liver enhancement is the mean RLE derived from the calculation according to the formula: Relative Enhancement (RLE) = (PostSI-PreSI)/PreSI, of all liver (9 segments including 4a and 4b) segments

*FF*, fat fraction is the mean value derived from the calculation according to the formula: [(SIin-SIopp)/2 × SIin] × 100. SIin and SIopp were liver parenchyma signal intensity on in-phase or opposed-phase images of all liver segments (9 segments including 4a and 4b)

*B*, unstandardized beta representing the slope of the line between the predictor variable and the dependent variable

In the multiple regression analysis using backward elimination, only fibrosis (Beta =  − 0.397, *p* = 0.040, Beta − 0.574, *p* ≤ 0.001) remained a significant predictor of NASH. Likewise, in the univariate analysis, FF was positively correlated with steatosis (Beta = 0.733, *p* = 0.001) and inflammation (Beta = 0.367, *p* = 0.012), but not with ballooning (Beta = 0.105, *p* = 0.485) or fibrosis (Beta = 0.069, *p* = 0.647). In the multiple regression analysis using backward elimination, only steatosis (Beta = 0.723, *p* ≤ 0.001) remained significant.

**Fig. 4:**
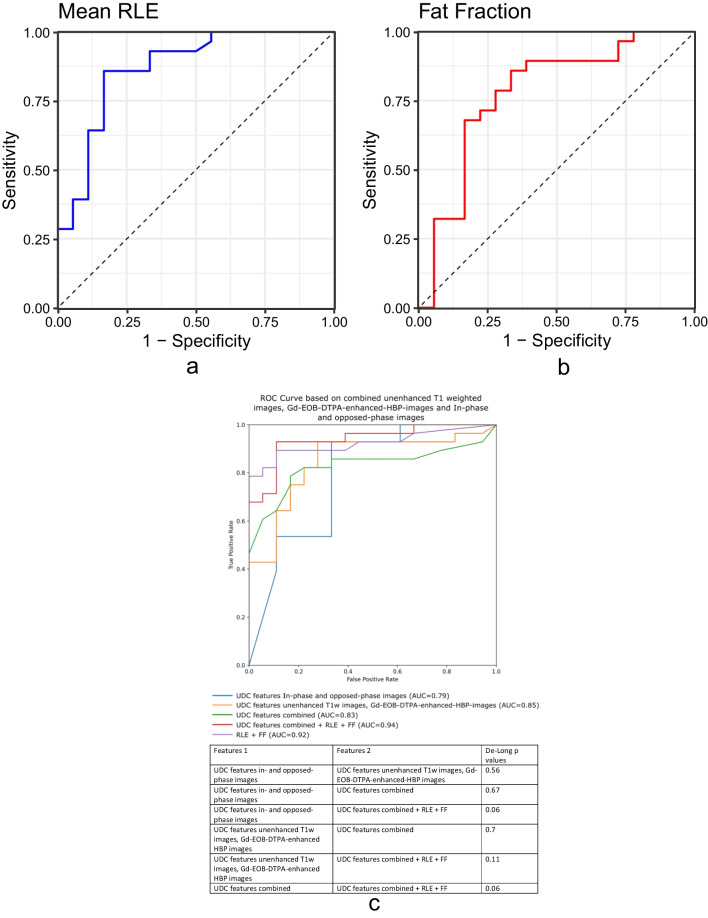
**a** ROC curve shows the diagnostic performance of MRI parameters using RLE (**a**) for unenhanced and T1-GA-HBP. The RLE was able to differentiate NASH from simple steatosis patients with an accuracy of 83.1% [AUROC = 0.808], a sensitivity of 85.7%, a specificity of 83.3%, a PPV of 88.9%, and an NPV of 78.9%, for a cutoff value of 1.20. **b** ROC curve shows the diagnostic performance of MRI parameters using in- and opposed-phase (CSI). The FF was able to differentiate NASH from simple steatosis patients with an accuracy of 78.3% [AUROC = 0.778], a sensitivity of 85%, a specificity of 66.7%, a PPV of 80.0%, and an NPV of 75.0%, for a cutoff value of 19.0. **c** Finally, we compared the efficacy of UDC using unenhanced T1 and T1-Gd-EOB-DTPA-HBP combined with CSI based on a Random Forest classifier, as well as RLE and FF using the DeLong method

ROC analysis of RLE, derived from unenhanced T1 and Gd-EOB-DTPA-HBP-T1 sequences, and FF quantification, yielded the diagnostic performance of differentiating between NASH and simple steatosis. For RLE, accuracy was 83.1% [AUROC = 0.808 (95% CI: 0.76–0.98)], sensitivity 85.7%, specificity 83.3%, PPV 88.9%, and NPV 78.9% (Fig. 4a).

The FF was able to differentiate NASH from simple steatosis patients with an accuracy of 78.3% [AUROC = 0.778 (95%CI 0.81–0.98)], a sensitivity of 85%, a specificity of 66.7%, a PPV of 80.0%, and a NPV of 75.0% (Fig. 4b).

### Results of combined UDC, RLE, and FF

Finally, with the DeLong method, we compared the efficacy of UDC, using unenhanced T1 and T1-Gd-EOB-DTPA-HBP, combined with CSI based on the Random Forest classifier, as well as RLE and FF [25]. The combined ROC curves, as well as the DeLong *p* values, can be found in Fig. 4c. While none of the *p* values reached the nominal threshold of statistical significance (*p* < 0.05), there was a trend showing an improvement in classification accuracy when combining RLE and FF with UDC features from both in-phase and opposed-phase images and unenhanced T1-weighted images/Gd-EOB-DTPA-enhanced HBP images against the UDC features alone (AUC UDC features combined + RLE + FF = 0.94, AUC UDC features combined = 0.83, DeLong *p* value = 0.06).

### Validation group

#### Characteristics

The validation group, retrospectively enrolled, consisted of 30 patients, *M* = 17 (56.7%), mean age of 57 years (range 30–78). There was no significant difference in age nor BMI between the simple steatosis and NASH subgroups. Among the laboratories, only the AST, AST/ALT, and NFS Score were significant (*p* ≤ 0.05) (Table 2S). The interval between MRI and biopsy, and MRI and laboratories was 1–3 months. Histologically, 13 and 17 patients were classified as simple steatosis and NASH, respectively.

All four histologic components of the SAF score, steatosis grade (*p* = 0.211), lobular inflammation (*p* < 0.001), ballooning (*p* = 0.062), and fibrosis (*p* ≤ 0.001), distinguished between simple steatosis and NASH subgroups. In particular, the majority of the NASH cohort had high inflammation and fibrosis scores but no difference on steatosis (Table 3S).

### Results of liver segmentation

For the validation cohort, we randomly sampled 4 of the 30 patients and created ground truth labels for the evaluation of the segmentation accuracy. The results from validation cohort (Dice: 0.956, recall: 0.956, precision: 0.955) were similar to the results on the T1-Gd-EOB-DTPA-HBP sequences from the derivation liver cohort.

### Results of unsupervised deep clustering (UDC)

Overall, for the validation group (*p* ≤ 0.001), we found features that distinguished NASH from simple steatosis using Student’s *t*-test (Table 4S). Again, using results from Random Forest regression to link MRI and the four histologic features in the validation group, UDC could differentiate between low- (grade < 3) and high-grade fibrosis (*p* < 0.001), and also characterize different grades of lobular inflammation (*p* = 0.04) and ballooning (*p* < 0.001).

In addition, Random Forest classifier was able to differentiate NASH from simple steatosis patients with an accuracy of 83.3% [AUROC = 0.87], a sensitivity of 70.6%, a specificity of 100%, a PPV of 100%, and a NPV of 72.2% (Fig. 3aS).

In the validation group, UDC signatures derived from CSI (T1-weighted chemical shift imaging) were able to differentiate between NASH and simple steatosis using Student’s *t*-test, *p* < 0.05 (Table 4S). Using Random Forest regression, UDC based on CSI could characterize different grades of lobular inflammation (*p* = 0.013) and ballooning (*p* < 0.001), and furthermore was able to differentiate NASH from simple steatosis patients with an accuracy of 43.3% [AUROC = 0.27], a sensitivity of 5.9%, a specificity of 92.3%, a PPV of 50%, and an NPV of 42.9% (Fig. 3bS). The Random Forest classifier, based on unenhanced T1 and T1-Gd-EOB-DTPA-HBP combined with CSI, could differentiate NASH from simple steatosis patients with an accuracy of 86.7% [AUROC = 0.88], a sensitivity of 76.5%, a specificity of 100%, a PPV of 100%, and a NPV of 76.5%. The combined ROC curve is depicted in Fig. 3cS.

### Results of MR-derived measurements (RLE and FF)

MRI parameters, including RLE and FF derived from the same images as those used in the UDC, again differed significantly between NASH and simple steatosis patients for both readers, with excellent inter-reader agreement for the measurements and high ICC (0.8–0.9) values (Table 4S).

Univariate and multivariate analyses of the relationship between RLE, FF, and histopathologic parameters are summarized in Table 5S. RLE was negatively correlated only with lobular inflammation (Beta =  − 0.410,* p* = 0.025), and degree of fibrosis (Beta =  − 0.574, *p* =  < 0.001), but not with the ballooning (Beta =  − 0.205, *p* = 0.277) nor liver steatosis (Beta =  − 0.005, *p* = 0.977) severity. Multiple regression found only fibrosis (Beta − 0.574, *p* ≤ 0.001) was a significant predictor of NASH.

Similarly, in univariate analysis, FF was negatively correlated with inflammation (Beta =  − 0.372, *p* = 0.043) and fibrosis (Beta =  − 0.366, *p* = 0.047). But on multiple regression, FF negatively correlated significantly only with inflammation (Beta = 0.476, *p* = 0.012).

The diagnostic performance of RLE and CSI for the differentiation between NASH and simple steatosis was evaluated using ROC analysis. For the RLE, the accuracy was 86.7% [AUROC = 0.90 (95% CI: 0.79–1)], sensitivity 88.2%, specificity 84.6%, PPV 88.2%, and NPV 84.6% (Fig. 4aS). For FF, the accuracy was 66.7% [AUROC = 0.73 (95% CI: 0.57–0.90)], sensitivity 41.1%, specificity 100%, PPV 100%, and NPV 56.5% (Fig. 4bS).

### Results of combined UDC, RLE, and FF

Finally, with the DeLong method, we compared the efficacy of UDC, using unenhanced T1 and T1-Gd-EOB-DTPA-HBP, combined with CSI based on Random Forest classifier, as well as RLE and FF [25]. The combined ROC curves, and the DeLong *p* values, can be found in Fig. 4cS. As with the derivation group, we also observed a trend showing an improvement in classification accuracy when combining RLE and FF with UDC features from chemical shift images and unenhanced T1-weighted images/Gd-EOB-DTPA-enhanced HBP images against the UDC features combined = 0.88, DeLong *p* value = 0.09.

## Discussion

Using histopathology as gold standard, our prospective data from the derivation group showed that, based upon identical MRI sequences, i.e., unenhanced T1- and Gd-EOB-DTPA-enhanced T1-weighted images (T1-Gd-EOB-DTPA-HBP), as well as CSI, i.e., in- and opposed-phase sequences, we could distinguish simple steatosis from NASH by applying two independent methods. These results were confirmed in the validation group. The first approach used unsupervised deep clustering (UDC) to derive MR imaging features, with a Random Forest model to separate simple steatosis from NASH. UDC, a relatively new method based upon deep clustering networks (DCN), links MRI texture patterns to histologic features [17]. The second approach relied upon fat fraction (FF) quantification and mean RLE calculation, i.e., liver parenchymal signal intensities, which have proven utility in NAFLD, as our results corroborated [15] [23, 26, 27]. The RLE was significantly higher in simple steatosis versus NASH cohorts in both derivation and validation groups, with a defined cutoff level of ≤ 1 [15, 23]. Furthermore, our readers also calculated significantly higher mean FF for NASH compared to simple steatosis patients in the derivation group [23], but the results were reversed for the validation group, reflecting the described mechanisms and sequelae of NAFLD [28]. Thus, our study confirmed the role of RLE, FF, and UDC in the diagnostic workup of NAFLD.

Interestingly, RLE based on unenhanced T1 and T1-Gd-EOB-DTPA-HBP, and FF based on CSI, had accuracies similar to those of UDC for separating NASH from simple steatosis. More strikingly, by employing UDC based on unenhanced T1 and T1-Gd-EOB-DTPA-HBP, we found not only that features distinguished NASH from simple steatosis, but also that the Random Forest classifier technique could also predict variables that were able to distinguish low- versus high-grade steatosis, low-grade versus high-grade fibrosis, and even grades of lobular inflammation and ballooning. On the contrary, when constructing the Random Forest classifier based on CSI-derived UDC data, only low- versus high-grade steatosis and inflammation severity could be distinguished, but not fibrosis severity or hepatocyte ballooning grade, confirmed in the validation group.

Similarly, on multivariate regression analysis of CSI-based FF, only steatosis remained an independent predictor of NASH, while ballooning, inflammation, and even fibrosis were eliminated in the derivation group. Furthermore, multivariate analysis of RLE based on unenhanced T1 and T1-Gd-EOB-DTPA-HBP images proved that only fibrosis was an independent histopathologic predictor of NASH, with all other components eliminated in this model.

To explain differences in the degree of steatosis between the derivation and validation groups, it should be acknowledged that NASH is caused by lipotoxicity from excess free lipid species (e.g., free fatty acids, ceramides) and not triglycerides per se. Therefore, there is no compelling correlation with the degree of steatosis or triglyceride content which may be considered a bystander rather than cause of lipotoxicity [29]. In line with the concept of lipid partitioning, in rodent models, retention of potentially toxic lipid species within otherwise inert lipid droplets can paradoxically protect the liver from lipid-induced hepatic insulin resistance by preventing activation of protein kinase C [28].

It is important to bear in mind that, of the four histologic variables, fibrosis has proven to be the best predictor of NASH outcome [30–33]. Whether or not there was any correction for confounders, an analysis of over 4,000 patients found that fibrosis stage correlates not only with liver-related morbidity and mortality, but even also with all-cause mortality [30]. Thus, to avert poor outcomes, any NAFLD patient with severe fibrosis should be closely monitored [1, 34].

Our results show that RLE is a robust method for separating simple steatosis from NASH, having both relatively high accuracy and accurate grading of fibrosis. Furthermore, a cutoff value of ≤ 1.0 has already been established for this purpose [15, 23]. However, UDC, particularly using unenhanced T1 and T1- Gd-EOB-DTPA-HBP, seems to be an even stronger predictor since it was able to detect and stage all four histologic features of NAFLD. At the same time, UDC segments the liver into areas of tissue comparable to that occurring across NAFLD individuals. Therefore, UDC may shed light on steatosis, inflammation, ballooning, and fibrosis and their response to therapeutic interventions, including diet and medication. This may be beneficial in longitudinal clinical studies of NAFLD patients.

According to our results, data derived from Gd-EOB-DTPA-HBP-enhanced MRI can reliably stage NAFLD, and predict fibrosis with RLE or all-histologic NASH components using UDC. CSI had less merit since it could only predict steatosis grade which, although helpful in diagnosing NAFLD, fails to inform about the prognosis and severity of the disease.

Generally, there are two systems for semiquantitative assessment or grading of NAFLD. The first is the NAFLD Activity Score (NAS) from the NASH CRN [35]. Its criteria were established using the Brunt classification, including steatosis (0–3), lobular inflammation (0–3), hepatocyte ballooning (0–2), and fibrosis (0–4) [36]. The second is the Steatosis Activity Fibrosis (SAF) score from the European Fatty Liver Inhibition of Progression Consortium [20]. We used the latter because, although the likelihood of NASH increases with NAS, there exists a wide gray zone (NAS 3–4) where NASH may or may not be present [20]. The SAF score is a simple scoring system that seems more relevant than simply dichotomizing cases according to the presence or absence of NASH [20]. Nevertheless, it is well-known that biopsy is prone to sampling error and interobserver variability in histologic grading of liver biopsies with any scoring system [6, 37]. This may be one reason why the AUROC for most validated panels, including the UDC, RLE, and FF, is in the 0.7–0.85 range and not higher. The shortfall is not these diagnostic tools, but rather the overlap of histologic severity of the four variables meant to separate NASH from simple steatosis. In addition, SAF and CRN scoring are less sensitive to histologic alterations than quantitation, which estimates only lobular inflammation rather than both lobular and portal inflammation, as with UDC, RLE, or FF [38]. There is also an overlap between the histologic ballooning score and ballooning quantitation using UDC and RLE, probably because pathologists rely more heavily on the quality rather than quantity. Finally, these imaging algorithms define fat percentage as a proportion of steatosis within the whole tissue area, rather than purely within hepatic cells as does a pathologist, making it subject to sampling variability. Therefore, a prospective study designed to directly compare UDC features derived from MRI to deep learning features derived from histopathology of the biopsy specimen may yield better results.

We have to acknowledge several limitations. Although we only had 46 patients in the derivation group, which limits the generalizability of our results, our independent validation group confirmed these results. Moreover, because clustering was done at the patch level rather than at the patient level, the sample size is 50,000 patches. Thus, the actual sample size is much larger than it appears. Furthermore, overfitting is reduced by using Random Forest classifier with 10 and 20 vector features, respectively. The quality of the data is supported by the fact that our validation group confirmed our initial findings. Regarding assessment of FF, a multi-echo technique would have provided more information for UDC rather than CSI, i.e., dual-echo in- and opposed-phase. However, because none of our NAFLD cohort had detectable hepatic iron, a known confounder that can underestimate FF, the dual-echo technique may have been sufficient, even if not as ideal as the multi-echo technique [39].

We used two different statistical methods, namely regression analysis for RLE and FF as each had only a single trait, they required a feature vector with only one degree of freedom. However, because UDC assessed several characteristics, we used a Random Forest classifier where the feature vector had several degrees of freedom. We suggest caution in quantitative comparisons between these methods and consider the results as exploratory. Lastly, this study is cross-sectional and does not provide evidence about the longitudinal benefit of MRI clinical prediction rules in detecting changes in NAFLD patients. Therefore, further prospective studies using AI-based computational analysis on both MRI, and histopathology specimen might further inform the relationship between micro- and macro-scale features.

In conclusion, two different techniques, UDC approach and imaging parameters (RLE and FF), could independently discriminate between NASH and simple steatosis based on identical data derived from unenhanced T1 and T1-Gd-EOB-DTPA-HBP MR images, as well as CSI. The UDC approach was comparable and proved able to predict all NAFLD components using unenhanced T1 and T1-Gd-EOB-DTPA-HBP images. The similarity of results between the derivation and validation groups confirms the robustness of this method.

Importantly, UDC does not require manual annotation of ROIs during evaluation, and is thus independent of operator bias and experience. The results indicate that machine learning approaches can identify predictive MRI patterns related to histopathology-derived parameters. This potentially allows their use to expand our vocabulary of imaging patterns, and generate hypotheses regarding their relationship to disease.

### Supplementary information

Below is the link to the electronic supplementary material.

Supplementary file1 (PDF 1.68 MB)

## Abbreviations

ALBI score: Albumin-bilirubin score
APRI score: Aspartate aminotransferase to platelet ratio index score
CSI: Chemical shift imaging
DCN: Deep clustering network
FF: Fat fraction
FIB-4 score: Fibrosis-4 score
Gd-EOB-DTPA-MRI: Gd-EOB-DTPA-enhanced MRI
NAFLD: Non-alcoholic fatty liver disease
NASH: Non-alcoholic steatohepatitis
NFS: NAFLD fibrosis score
PDFF: Proton density fat fraction
PostSI: Post-contrast signal intensity
PreSI: Pre-contrast signal intensity
RLE: Relative liver enhancement
SAF score: Steatosis Activity Fibrosis score
SIin: SI in-phase
SIout: SI out-of-phase
T1-Gd-EOB-DTPA-HBP: T1-weighted Gd-EOB-DTPA-enhanced hepatobiliary phase
UDC: Unsupervised deep clustering

## Declarations

### Guarantor

The scientific guarantor of this publication is Dr. Ahmed Ba-Ssalamah.

### Statistics and biometry

Dr. Michael Weber kindly provided statistical advice for this manuscript.

### Study subjects or cohort overlap

Some study subjects or cohorts have been previously reported in “Application of localized 31P MRS saturation transfer at 7 T for measurement of ATP metabolism in the liver: reproducibility and initial clinical application in patients with non-alcoholic fatty liver disease” published in Eur Radiol (2014) 24: 1602–1609. This study has nothing to do with RLE, FF, and UDC or AI.

### Methodology

• prospective

• observational

• performed at one institution

## References

[1] Chalasani N, Younossi Z, Lavine JE (2018). The diagnosis and management of nonalcoholic fatty liver disease: practice guidance from the American Association for the Study of Liver Diseases. Hepatology.

[2] European Association for the Study of the L, European Association for the Study of D, European Association for the Study of O (2016). EASL-EASD-EASO Clinical Practice Guidelines for the management of non-alcoholic fatty liver disease. J Hepatol.

[3] Singh S, Allen AM, Wang Z, Prokop LJ, Murad MH, Loomba R (2015) Fibrosis progression in nonalcoholic fatty liver vs nonalcoholic steatohepatitis: a systematic review and meta-analysis of paired-biopsy studies. Clin Gastroenterol Hepatol 13:643–654 e641–649; quiz e639–640 10.1016/j.cgh.2014.04.014 PMC4208976 24768810

[4] Mortality GBD, Causes of Death C,  (2016). Global, regional, and national life expectancy, all-cause mortality, and cause-specific mortality for 249 causes of death, 1980–2015: a systematic analysis for the Global Burden of Disease Study 2015. Lancet.

[5] Wu T, Gao X, Chen M, van Dam RM (2009). Long-term effectiveness of diet-plus-exercise interventions vs. diet-only interventions for weight loss: a meta-analysis. Obes Rev.

[6] Sumida Y, Nakajima A, Itoh Y (2014). Limitations of liver biopsy and non-invasive diagnostic tests for the diagnosis of nonalcoholic fatty liver disease/nonalcoholic steatohepatitis. World J Gastroenterol.

[7] Ratziu V, Charlotte F, Heurtier A (2005). Sampling variability of liver biopsy in nonalcoholic fatty liver disease. Gastroenterology.

[8] Utsunomiya T, Shimada M, Hanaoka J (2011). Possible utility of MRI using Gd-EOB-DTPA for estimating liver functional reserve. J Gastroenterol.

[9] Vilar-Gomez E, Chalasani N (2018). Non-invasive assessment of non-alcoholic fatty liver disease: clinical prediction rules and blood-based biomarkers. J Hepatol.

[10] Zhou JH, Cai JJ, She ZG, Li HL (2019). Noninvasive evaluation of nonalcoholic fatty liver disease: current evidence and practice. World J Gastroenterol.

[11] Springer F, Machann J, Schwenzer NF (2010). Quantitative assessment of intrahepatic lipids using fat-selective imaging with spectral-spatial excitation and in-/opposed-phase gradient echo imaging techniques within a study population of extremely obese patients: feasibility on a short, wide-bore MR scanner. Invest Radiol.

[12] Yokoo T, Serai SD, Pirasteh A (2018). Linearity, bias, and precision of hepatic proton density fat fraction measurements by using MR imaging: a meta-analysis. Radiology.

[13] Runge JH, Smits LP, Verheij J (2018). MR spectroscopy-derived proton density fat fraction is superior to controlled attenuation parameter for detecting and grading hepatic steatosis. Radiology.

[14] Costa-Silva L, Ferolla SM, Lima AS, Vidigal PVT, Ferrari TCA (2018). MR elastography is effective for the non-invasive evaluation of fibrosis and necroinflammatory activity in patients with nonalcoholic fatty liver disease. Eur J Radiol.

[15] Bastati N, Feier D, Wibmer A (2014). Noninvasive differentiation of simple steatosis and steatohepatitis by using gadoxetic acid-enhanced MR imaging in patients with nonalcoholic fatty liver disease: a proof-of-concept study. Radiology.

[16] Caussy C, Reeder SB, Sirlin CB, Loomba R (2018). Noninvasive, quantitative assessment of liver fat by MRI-PDFF as an endpoint in NASH Trials. Hepatology.

[17] Perkonigg M, Sobotka D, Ba-Ssalamah A, Langs G (2019) UUnsupervised deep clustering for predictive texture pattern discovery in medical images. 33rd Conference on Neural Information Processing Systems (NeurIPS 2019), Vancouver, Canada. 10.48550/arXiv.2002.03721

[18] Bo Yang XF, Nicholas D Sidiropoulos, and Mingyi Hong (2017) Towards k-means-friendly spaces:simultaneous deep learning and clustering. . In Proceedings of the 34th International Conference on Machine Learning-Volume 70, pages 3861–3870

[19] Breiman L (2001). Random forests Machine learning.

[20] Bedossa P, Poitou C, Veyrie N (2012). Histopathological algorithm and scoring system for evaluation of liver lesions in morbidly obese patients. Hepatology.

[21] Ronneberger O, Fischer, P., & Brox, T. (2015) U-net: Convolutional networks for biomedical image segmentation. . In International Conference on Medical image computing and computer-assisted intervention 234–241

[22] Yang B, Fu X, Sidiropoulos ND, Hong M (2017) Towards K-means-friendly spaces: simultaneous deep learning and clustering. ICML'17: Proceedings of the 34th International Conference on Machine Learning - Volume 70:3861–3870

[23] Wu Z, Matsui O, Kitao A (2013). Usefulness of Gd-EOB-DTPA-enhanced MR imaging in the evaluation of simple steatosis and nonalcoholic steatohepatitis. J Magn Reson Imaging.

[24] Koo TK, Li MY (2016). A guideline of selecting and reporting intraclass correlation coefficients for reliability research. J Chiropr Med.

[25] DeLong ER, DeLong DM, Clarke-Pearson DL (1988) Comparing the areas under two or more correlated receiver operating characteristic curves: a nonparametric approach. Biometrics 44(3):837-845 3203132

[26] Tsuda N, Okada M, Murakami T (2007). Potential of gadolinium-ethoxybenzyl-diethylenetriamine pentaacetic acid (Gd-EOB-DTPA) for differential diagnosis of nonalcoholic steatohepatitis and fatty liver in rats using magnetic resonance imaging. Invest Radiol.

[27] Tsuda N, Matsui O (2011). Signal profile on Gd-EOB-DTPA-enhanced MR imaging in non-alcoholic steatohepatitis and liver cirrhosis induced in rats: correlation with transporter expression. Eur Radiol.

[28] Loomba R, Friedman SL, Shulman GI (2021). Mechanisms and disease consequences of nonalcoholic fatty liver disease. Cell.

[29] Neuschwander-Tetri BA (2010). Hepatic lipotoxicity and the pathogenesis of nonalcoholic steatohepatitis: the central role of nontriglyceride fatty acid metabolites. Hepatology.

[30] Taylor RS, Taylor RJ, Bayliss S (2020). Association between fibrosis stage and outcomes of patients with nonalcoholic fatty liver disease: a systematic review and meta-analysis. Gastroenterology.

[31] Younossi Z, Anstee QM, Marietti M (2018). Global burden of NAFLD and NASH: trends, predictions, risk factors and prevention. Nat Rev Gastroenterol Hepatol.

[32] Ekstedt M, Hagstrom H, Nasr P (2015). Fibrosis stage is the strongest predictor for disease-specific mortality in NAFLD after up to 33 years of follow-up. Hepatology.

[33] Angulo P, Kleiner DE, Dam-Larsen S (2015). Liver fibrosis, but no other histologic features, is associated with long-term outcomes of patients with nonalcoholic fatty liver disease. Gastroenterology.

[34] Dulai PS, Singh S, Patel J (2017). Increased risk of mortality by fibrosis stage in nonalcoholic fatty liver disease: systematic review and meta-analysis. Hepatology.

[35] Kleiner DE, Brunt EM, Van Natta M (2005). Design and validation of a histological scoring system for nonalcoholic fatty liver disease. Hepatology.

[36] Brunt EM, Kleiner DE, Wilson LA, Belt P, Neuschwander-Tetri BA, Network NCR (2011). Nonalcoholic fatty liver disease (NAFLD) activity score and the histopathologic diagnosis in NAFLD: distinct clinicopathologic meanings. Hepatology.

[37] Mehta SH, Lau B, Afdhal NH, Thomas DL (2009). Exceeding the limits of liver histology markers. J Hepatol.

[38] Forlano R, Mullish BH, Giannakeas N (2019). High-throughput, machine learning-based quantification of steatosis, inflammation, ballooning, and fibrosis in biopsies from patients with nonalcoholic fatty liver disease. Clin Gastroenterol Hepatol.

[39] Satkunasingham J, Besa C, Bane O (2015). Liver fat quantification: comparison of dual-echo and triple-echo chemical shift MRI to MR spectroscopy. Eur J Radiol.

