# Supplementary material for: Correlation of histologic, imaging, and artificial intelligence features in NAFLD patients, derived from Gd-EOB-DTPA-enhanced MRI: a proof-of-concept study
Source: Eur Radiol. 2023 Jun 26;33(11):7729–43. doi: 10.1007/s00330-023-09735-5 (PMC10598123; doi:10.1007/s00330-023-09735-5)
Supplement: Supplementary file 1 — Supplementary file1 (PDF 1.68 MB) [file 330_2023_9735_MOESM1_ESM.pdf]

Table 1S. Validation group. MR protocol with exam parameter

Sequences	Slice orientation	Matrix	Voxel mm	FOV mm	SL mm	Phase Direction	TR Ms	TE ms	FA Degree	Time s
GRE T1 (2D flash) in-phase*	Axial	180x320	0.6x0.6x1.7	400	1.7-2	AP	170	2.5	20	21
GRE T1 (2D flash) opposed-phase*	Axial	165x320	0.6x0.6x1.7	400	1.7-2	AP	170	4.3	20	21
T1 VIBE FatSat unenhanced*	Axial	165x320	0.6x0.6x1.7	400	1.7-2	AP	4.4	1.33	20	21
T1 VIBE FatSat gadoxetic-enhanced (arterial and portal-venous)	Axial	165x320	0.6x0.6x1.7	400	1.7-2	AP	4.4	1.33	20	21x3
T1 VIBE FatSat 5 min post contrast (transitional)	Axial	165x320	0.6x0.6x1.7	400	1.7-2	AP	4.4	1.33	20	21
DWI TSE-EP/ADC	Axial	134x134	1.5x1.5x5	400	5	AP	3400	38	90	6.25
T2 HASTE fs	Axial	176x320	1.3x1.3x5	400	5	AP	1500	150	150	1.24
T2 HASTE	Coronal	256x256	1.6x1.6x5	400	5	RL	1160	150	143	1.01

T1 VIBE FatSat 20 min post contrast (HBP)*	Axial	165x320	0.6x0.6x1.7	400	1.7-2	AP	4.4	1.33	20	21
T1 VIBE FatSat 20 min post contrast (HBP)	Coronal	243x320	1.4x1.4x1.5	450	1.5-2	RL	4.5	1.3	20	18

* evaluated for the study, HASTE = Half-Fourier Acquisition Single-shot Turbo spin Echo imaging; DWI TSE-EP/ADC = Diffusion-Weighted Imaging Turbo Spin Echo-Echo-Planar; MRCP= Magnetic Resonance Cholangiopancreatography; MIP=Maximum Intensity Projection; GRE = Gradient echo, VIBE = Volumetric Interpolated Breath-hold Examination, FOV = Field of view, Voxel = Voxel size, SL = Slice thickness, TR = Repetition time, TE = Echo time, FA = Flip angle, Time = Acquisition time.

Table 2S. Validation group. Anthropometric, clinical and laboratory characteristics of 30 patients of the two groups of NAFLD, (simple steatosis, and NASH).

Parameter	Simple Steatosis (13 pats)	NASH (17 patients)	P Value
Age (years)			
All patients	57.9±12.9 (34-72.7)	57.2±16.3 (29.8-78.1)	0.892
Men	59.6±13.9 (34-72.7)	62.8±12.5 (45.6-78.1)	0.629
Women	52.4±8.2 (46.7-61.8)	53.2±18.0 (29.8-75.2)	0.942
Body mass index (kg/m2)	30.4±5.47 (20.7-40.1)	31.26±7.46 (19.6-49.31)	0.706
Alanine aminotransferase (ALT) (U/L)	43.1±26.0 (14-107)	56.6±42.5 (20-181)	0.302
Aspartate aminotransferase (AST) (U/L)	31.2±14.2 (16-62)	62.3±33.8 (125-144)	**0.003**
g-glutamyl transpeptidase (GGT) (U/L)	149.2±188.3 (32-731)	209.0±233.6 (17-703)	0.452
Alkaline phosphatase (ALT) (U/L)	82.6±34.01 (41-167)	106.2±37.4 (47-173)	0.088
Total proteins (g/l)	69.1±8.5 (46.7-78.3)	67.8±6.3 (55.9-75.1)	0.373
Albumin (g/l)	44.3±6.5 (26.5-49.0)	40.4±5.9 (25.3-50.2)	0.110
Total bilirubin (mg/dL)	0.6±0.3 (0.13-1.4)	1.6±2.1 (0.31-8.9)	0.091

Triglycerides (mg/dL)	243.2±183.9 (62-786)	128.3±56.3 (77-263)	0.048	
HDLC (mg/dL)	33.0±26.1 (0-73)	53.3±19.8 (20-1ß3)	0.030	
Platelets (/mm^3)	202.6±53.9 (111-287)	170.1±95.8 (23-383)	0.285	
Glucose (mg/dL)	63.8±70.9 (0-228)	72.4±63.6 (0-184)	0.725	
AST/ALT ratio	0.86±0.45 (0.47-2.14)	1.32±0.63 (0.5-2.38)	**0.029**	
APRI_limit42male_35female -Score	0.37±0.17 (0.14-0.71)	1.75±3.08 (0.16-13.04)	0.093	
APRI_limit50male_35female -Score	0.31±0.14 (0.08-0.83)	1.47±2.58 (0.13-10.95)	0.098	
ALBI -Score	-2.6±0.5 (-2.9-1.16)	-2.24±0.48 (-2.96-1.0)	0.101	
NFS-Score	-52.9±10.4(-65.1-34.4)	-43.2±13.9 (-73.9--15.0)	**0.039**	
Fib-4-Score	0.27±0.23 (0.08-0.83)	0.81±1.09 (0.07-4.72)	0.071	

Note. Data are means and standard deviations with ranges in parentheses, except where indicated otherwise. To convert from units per liter to micrograms per liter, multiply by 0.0167. To convert from milligrams per deciliter (for bilirubin) to micromoles per liter, multiply by 17.104. To convert from milligrams per deciliter (for triglycerides) to millimoles per liter, multiply by 0.0113. To convert from milligrams per deciliter (for high-density lipoprotein cholesterol) to millimoles per liter, multiply by 0.0259. To convert from milligrams per deciliter (for glucose) to millimoles per liter, multiply by 0.0555.

Abbreviations: APRI: Aspartate aminotransferase to platelet ratio index, ALBI: Albumin-Bilirubin score, NFS: NAFLD Fibrosis Score, FIB-4 score: Fibrosis index based on 4 factor.

FIB-4 = Age (years)×AST (U/L)/[PLT(109/L)×ALT1/2 (U/L)].

NAFLD fibrosis score = -1.675 + 0.037 × age (year) + 0.094 × BMI (kg/m2) + 1.13 × IFG/diabetes (yes = 1, no = 0) + 0.99 × AST/ALT ratio - 0.013 × platelet count (×109/L) - 0.66 × albumin (g/dL).

ALBI score = (log10 bilirubin [µmol/L] × 0.66) + (albumin [g/L] × −0.0852).

APRI score = [(AST/upper limit of the normal AST range) X 100]/Platelet

Table 3S. Validation group. Histological characteristics of NAFLD (Simple steatosis vs. NASH) patients according to SAF score

	Histology parameters	Simple steatosis Patients (n=13)	NASH patients (n=17)	p
**Steatosis grade**				p 0.211
**1 (5-33%)**		7 (53.8%)	4 (23.5%)	
**2 (34-66%)**		4 (30.8 %)	10 (58.9%)	
**3 (>66%)**		2 (15.4%)	3 (17.6%)	
**ACTIVITY**	**Lobular inflammation**			**p<0.001**
	0 (none)	6 (46.2%)	0 (0 %)	
	1 (≤2 foci per 20x-magnification)	7 (53.8%)	9 (52.9%)	
	2 (>2 foci per 20x-magnification)	0 (0%)	8 (47.1%)	
	**Ballooning**			p= 0.062
	0 (none)	5 (38.5%)	1 (5.9%)	
	1 (slight)	5 (38.5%)	9 (52.9%)	

	2 (clear)	3 (23.1%)	7 (41.2%)	
**Fibrosis**				**p<0.001**
**0**		9 (69.2%)	0 (0%)	
**1a, b, c**		3 (23.1%)	1 (5.9%)	
**2**		0 (0%)	4 (23.5%)	
**3**		0 (0%)	4 (23.5%)	
**4**		1 (7.7%)	8 (47.1%)	

Note: Data are numbers of patients and numbers in parentheses are percentages, except where indicated otherwise.

NAFLD: Non-Alcoholic Fatty Liver Disease

SAF: Steatosis Activity Fibrosis

NASH: Non-Alcoholic Steatohepatitis

Table 4S. Validation group

MR Imaging and UDC parameters demonstrating the differences between simple steatosis and NASH of 30 patients with NAFLD according to the SAF score for both readers (R1 and R2) using a t-test.							
**Parameter**	**Simple Steatosis 13 patients R1**	**NASH 17 patients R1**	**†P Value**	**Simple Steatosis 13 patients R2**	**NASH 17 patients R2**	**†P Value**	**ICC**
UDC (unenhanced T1- and Gd-EOB-DTPA-T1-HBP)	0.30 ± 0.21	0.75 ± 0.28	**<0.001**				
UDC (CSI, in- and opposed-phase)	0.78 ± 0.16	0.54 ± 0.29	**<0.05**				
Mean signal intensity unenhanced T1	83.69±25.44	83.18±30.22	0.963	81.25±21.44	78.98±26.57	0.822	0.903
Mean signal intensity Gd-EOB-DTPA-T1-HBP	200.38±27.24	125.08±44.98	**<0.001**	196.28±29.09	112.03±61.85	**<0.001**	0.930
Mean relative liver enhancement (RLE)	1.58±0.78	0.75±0.57	**0.004**	1.60±0.71	0.85±0.23	**<0.001**	0.974
FF (PDFF/CSI, in- and opposed-phase)	22.54±7.08	21.35±3.15	0.011	22.77±4.32	21.53±3.17	0.027	0.896

*Data are means with standard deviations. † If the P value was less than the conventional level of .05, the corresponding variable was statistically significant and is written in bold type. RLE: relative liver enhancement, FF: fat fraction.

CSI: Chemical shift imaging dual echo: in-phase and out-of-phase.

UDC (unenhanced T1 & HBP): unsupervised deep clustering derived from unenhanced T1 and T1, 20 minutes after injection of Gd-EOB-DTPA acid in the hepatobiliary phase (HBP).

UDC (CSI, in- and opposed-phase): unsupervised deep clustering derived from chemical shift imaging (in-phase and out-of-phase).

Table 5S . Validation group. Correlation of conventional MR parameters using RLE/FF and Histologic Parameters according to Univariate and Multiple Regression Analysis for reader 1.

Parameter	Univariate					Multivariate				
**RLE**	B	P Value	Beta	95% CI		B	*P* Value	Beta	95% CI	
Steatosis	-0.005	0.977	-0. 005	-0.356	0.366					
Inflammation	-0.395	0.025	-0.410	-0.735	-0.055	-0.188	0.331	-0.195	-0.578	0.202
Ballooning	-0.187	0.277	-0.205	-0.533	0.159					
Fibrosis	-0.230	<0.001	-0.574	-0.357	-0.103	-0.230	**<0.001**	-0.574	-0.357	-0.103
**FF**	B	P Value	Beta	95% CI		B	*P* Value	Beta	95% CI	
Steatosis	0.351	0.309	0.192	-0.343	1.046					
Inflammation	-0.702	0.043	-0.372	-1.381	-0.023	-0.898	-0.012	-0.476	-1.581	-0.216
Ballooning	-0.159	0.639	-0.089	-0.8491	0.530					

Fibrosis	-0.287	0.047	-0.366	-0.571	-0.004	-0.203	0.214	-0.258	-0.530	0.124

Note: RLE: Relative liver enhancement is the mean RLE derived from the calculation according to the formula: Relative Enhancement (RLE) = (PostSI-PreSI)/PreSI, of all liver (9 segments including 4a and 4b) segments.

FF: Fat fraction is the mean value derived from the calculation according to the formula: [(SIin-SIopp)/2xSIin]x100. SIin and SIopp were liver parenchyma signal intensity on in-phase or opposed-phase images of all liver segments (9 segments including 4a and 4b).

If the P value is less than the conventional level of .05, the corresponding variable contributes significantly to the prediction of the dependent variable (RLE or FF).

In multiple regression analysis only liver fibrosis was significantly associated with the relative enhancement measurements (RLE) and only steatosis was significantly associated with fat fraction (FF).

B: Unstandardized beta representing the slope of the line between the predictor variable and the dependent variable

Supplementary Figures

Figure 3S)

ROC curves showing the random forest-based diagnostic performance of UDC for differentiating NASH from simple steatosis in the validation group, based on histology, using (a) unenhanced and T1-GA-HBP, (b) CSI, i.e., in-phase and opposed-phase and (c) combined unenhanced, T1-Gd-EOB-DTPA-HBP and CSI

Figure 3aS) The random forest classifier, based on (a) unenhanced and T1-GA-HBP, was able to differentiate NASH from simple steatosis in the validation group patients with an accuracy of 83.3% [AUROC=0.87], a sensitivity of 70.6%, a specificity of 100%, a PPV of 100%, and an NPV of 72.2%.

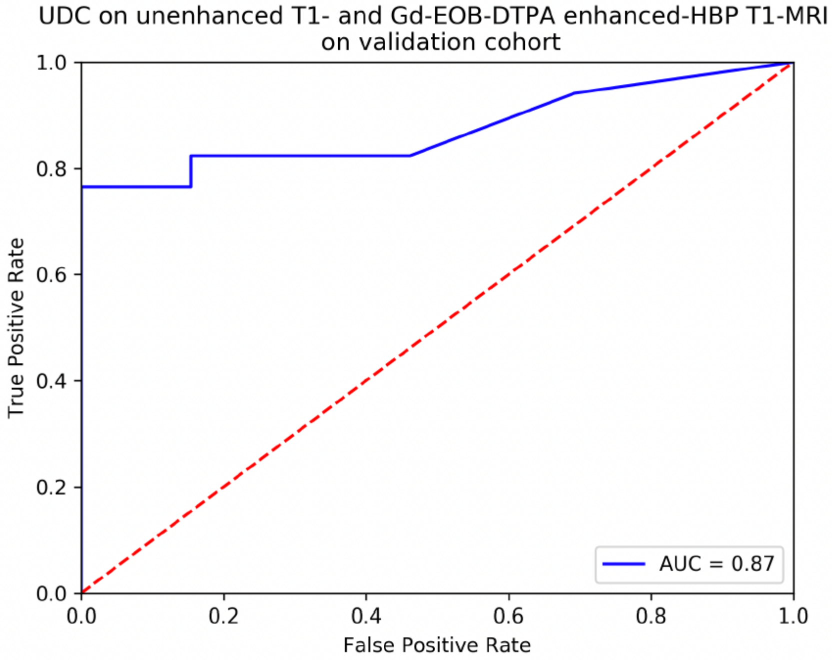

Figure 3bs)

The Random Forest classifier, based on (b) In- and opposed-phase (CSI), was able to differentiate NASH from simple steatosis in the validation group patients with an accuracy of 43,3% [AUROC=0.27], a sensitivity of 5.9%, a specificity of 92,3 %, a PPV of 50%, and an NPV of 42,9%.

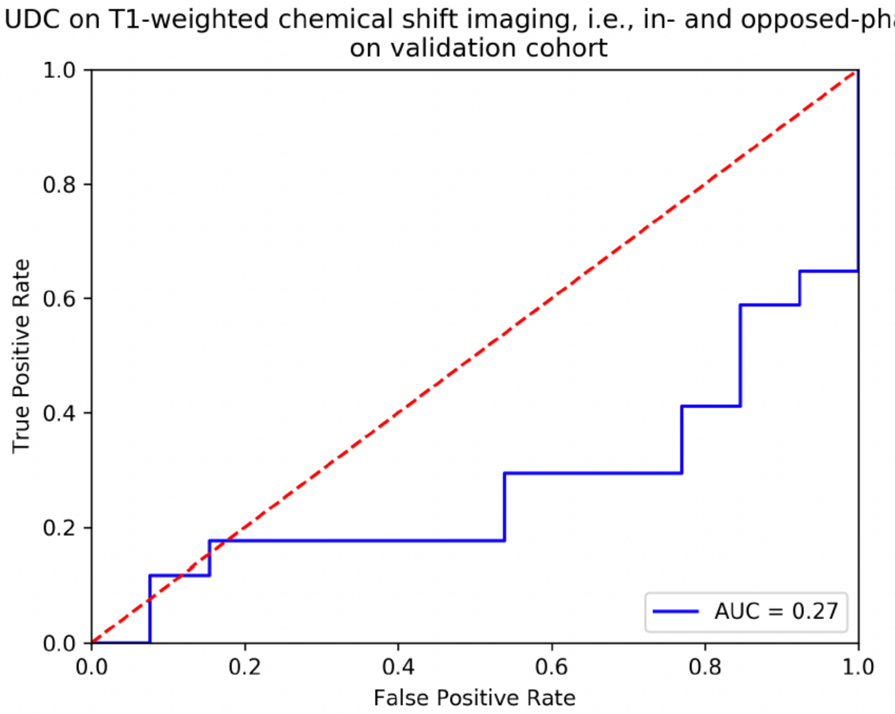

Figure 3cs)

The Random Forest classifier, based on unenhanced T1- and T1-Gd-EOB-DTPA-HBP combined with CSI, was able to differentiate NASH from simple steatosis in the validation group patients with an accuracy of 86.7% [AUROC=0.88], a sensitivity of 76.5%, a specificity of 100%, a PPV of 100% and a NPV of 76.5%

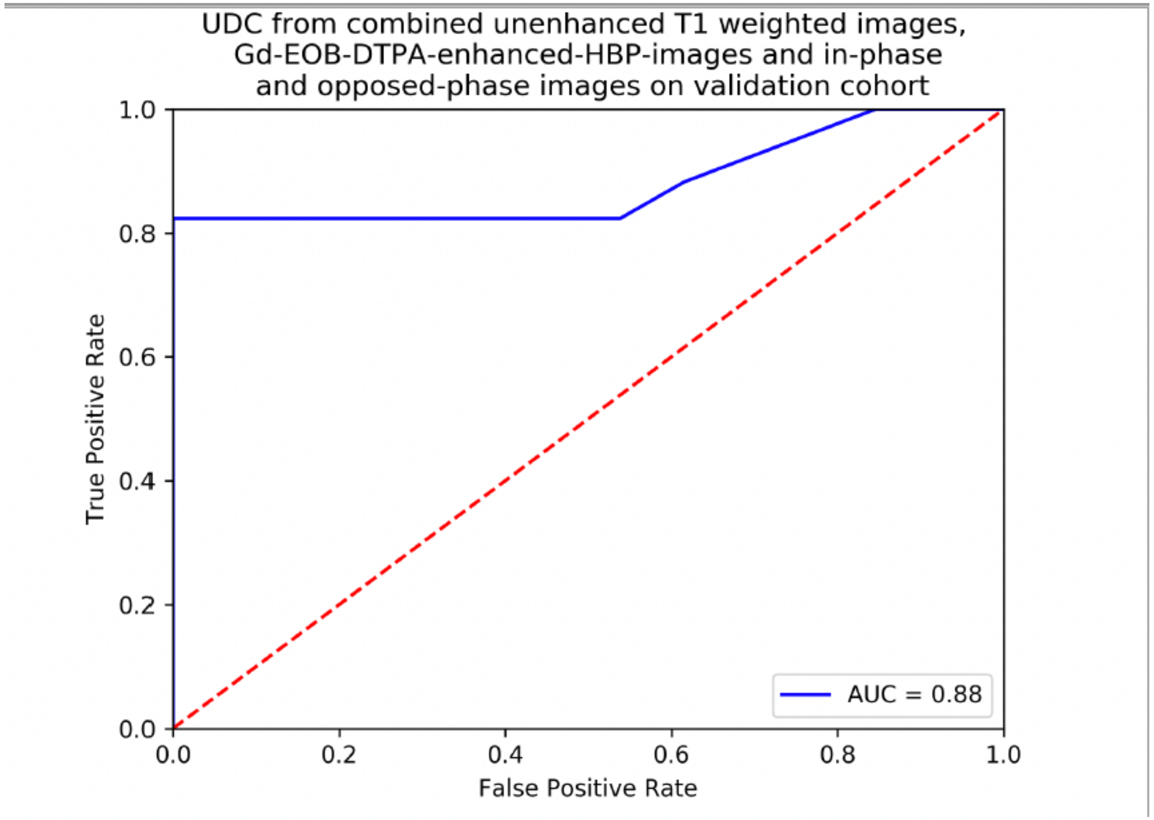

Figure 4S)

Results of Combined Unsupervised Deep-Clustering (UDC) and MR-derived Measurements (RLE and FF)

Figure 4aS)

ROC curve shows the diagnostic performance of MRI parameters using RLE (a) for unenhanced and T1-GA-HBP.

The RLE was able to differentiate NASH from simple steatosis patients with an accuracy of 86.7% [AUROC=0.90 (95% CI: 0.79-1)], a sensitivity of 88.2%, a specificity of 84%, a PPV of 88.2%, and an NPV of 84.6%.

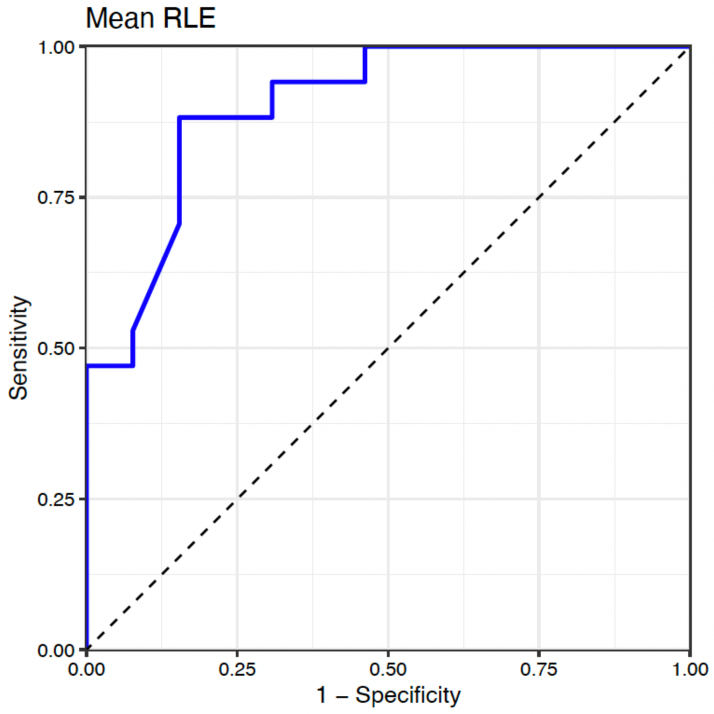

Figure 4bS)

ROC curve shows the diagnostic performance of MRI parameters using in- and opposed-phase (CSI). The FF was able to differentiate NASH from simple steatosis patients with an accuracy of 66.7% [AUROC=0.73], a sensitivity of 41.1%, a specificity of 100%, a PPV of 100%, and an NPV of 56.5%.

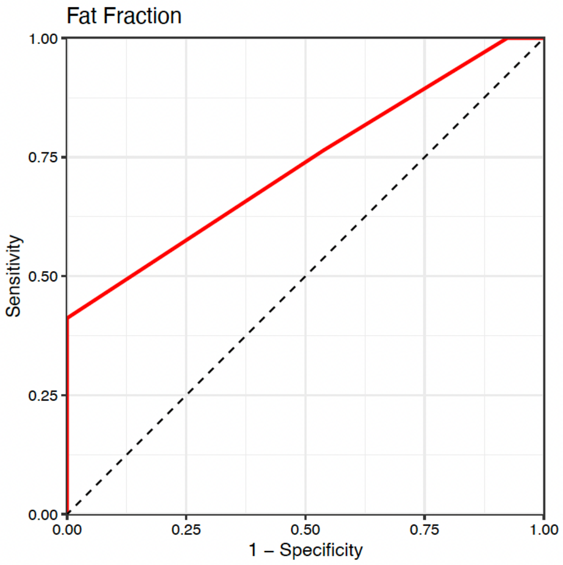

Figure 4cS)

Efficacy of UDC using unenhanced T1- and T1-Gd-EOB-DTPA-HBP combined with CSI based on Random Forest Classifier, as well as RLE and FF using the DeLong method on the validation cohort. AUC: 0.99, (DeLong p value = 0.09).

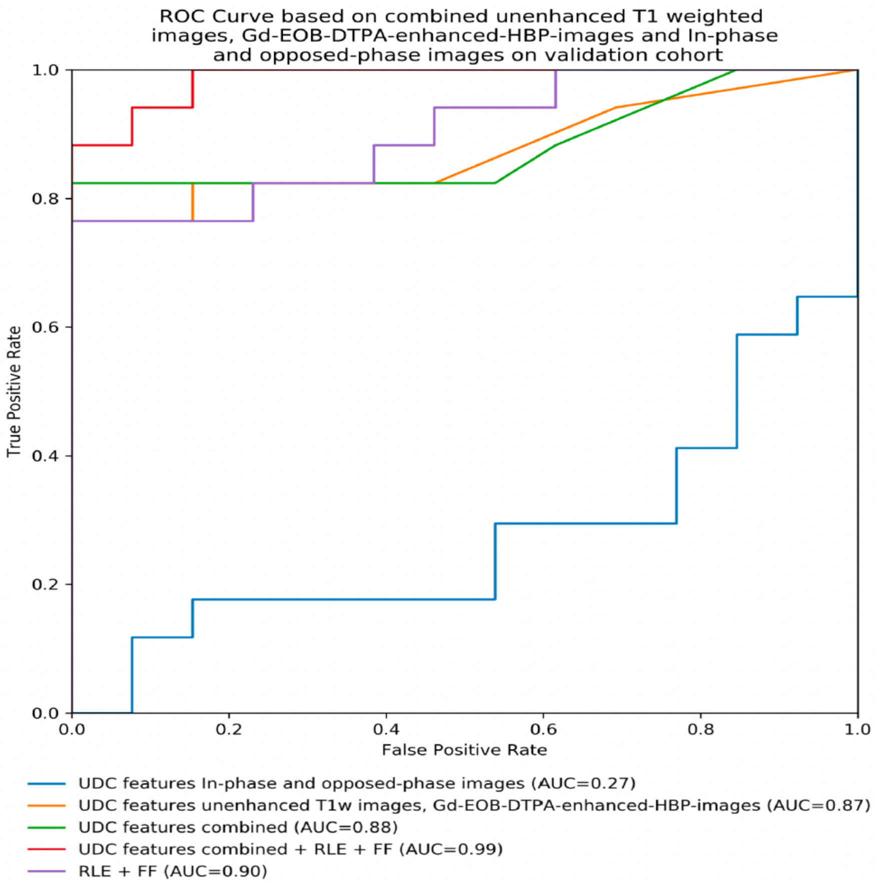

Features 1	Features 2	De-Long p values
UDC features In-phase and opposed-phase images	UDC features unenhanced T1w images, Gd-EOB-DTPA-enhanced-HBP-images	<0.01
UDC features In-phase and opposed-phase images	UDC features combined	<0.01
UDC features In-phase and opposed-phase images	UDC features combined + RLE + FF	<0.01
UDC features unenhanced T1w images, Gd-EOB-DTPA-enhanced-HBP-images	UDC features combined	0.80
UDC features unenhanced T1w images, Gd-EOB-DTPA-enhanced-HBP-images	UDC features combined + RLE + FF	0.06
UDC features combined	UDC features combined + RLE + FF	0.09
